# Periodization and Self-Regulation in Action Sports: Coping With the Emotional Load

**DOI:** 10.3389/fpsyg.2018.01652

**Published:** 2018-09-04

**Authors:** David Collins, Tom Willmott, Loel Collins

**Affiliations:** ^1^Institute of Coaching and Performance, University of Central Lancashire, Preston, United Kingdom; ^2^Snowsport New Zealand, Wanaka, New Zealand

**Keywords:** self-regulation, extreme sports, coaching practice, snow sports, Olympic sports

## Abstract

Action sports usually include some danger and personal challenge. The levels of both are often further increased when the sport is placed in a competitive environment. In this paper, we consider the Olympic disciplines of freeskiing and snowboarding in park and pipe. We consider some pertinent theoretical perspectives, then offer some insights on their operation using a range of data from ongoing research and support work. Finally, we offer a number of practical steps which can be taken to optimize performance, both in learning and practicing new tricks and in executing them under the pressures of competition.

## Introduction

Extreme or action sports are frequently defined as activities where the likely outcome of a mismanaged mistake or accident is death (Brymer and Schweitzer, 2017a,b; Frühauf et al., 2017). Action sports originate from a range of sources. Some, such as rope free climbing, big wave surfing, mountaineering above 8000 m, waterfall kayaking, extreme skiing and snowboarding stem from adventure sports (Collins and Collins, 2012). Increasingly, however, others derive from more traditional sports. Interestingly, many action sports have multiple versions many of which are competitive and now organized similarly to mainstream sport while others retain their non-competitive roots. For example, surfing has an international governing body and is now a competitive sport, climbing has a competitive dimension, and both are now Olympic sports whist also retaining a non-competitive element. We suggest that freeskiing and snowboarding have a comparatively longer history of crossing the competitive/non-competitive divide that possible stems from its origins as an action sport (Ojala and Thorpe, 2015; Thorpe, 2016). In contrast, parkour, with a similar origin, acts as more recent example that is now recognized as a sport with its own governing body and regulations.

Risk is common to many action sports. When the sport moves “into the mainstream” (cf. Willmott and Collins, 2015) the challenges from risk (e.g., “will I be able to learn/complete this move safely?”) are further complicated by those of competition (e.g., “will I win/do myself justice”), resulting in an increased level of psychological load for performers. The Olympic disciplines of Park and Pipe (hereafter P&P) are one such sport. Involving both skiers and snowboarders, the disciplines require participants to master and perform potentially dangerous tricks of up to four full rotations with triple twists; the current top end in this rapidly progressing sport. Indeed, these high levels of personal risk, combined with the tight social structures and ego commitment to the role of being a P&P athlete, mean that all levels face some degree of challenge.

As such, it is crucial for coaches and psychologists involved to understand and mitigate the negative impact and implications of this emotional load. Accordingly, in this paper we firstly consider some theoretical constructs which can usefully be applied to understanding and parameterizing the issue. This is followed by some exemplar data from our work in the field that help to establish how the theory applies. Finally, we present some systems and ideas which can be used to counter or control the impact on both performance and the individual.

## Theoretical Perspectives

After careful consideration, we would highlight two major theories which apply in P&P. These are Resource Depletion Theory (RDT), as placed within work on self-control and self-regulation or SR (e.g., Vohs et al., 2012) and the almost ubiquitous if ill-defined ideas of Mental Toughness (MT; Jones et al., 2002). Other ideas are apparent but would seem of questionable applicability for the P&P environment. For example, the “adrenaline junky” idea which has led some to see action sports participants as almost addicted to the “high” of risk (e.g., Buckley, 2012; Heirene et al., 2016). As highlighted by several recent studies (e.g., Willmott and Collins, 2017), elite P&P athletes are certainly positive about the lifestyle and achievement but seem less so about the risks! Indeed, their reported perceptions of risk as a severe challenge and a factor to be controlled would otherwise seem a contradiction. Certainly, recent research attests to the variation in participant motives across extreme sports (Barlow et al., 2013) so we are comfortable staying with the RDT/MT focus.

Work on self-control and SR has shown the wide-ranging issues which can occur for individuals low in this key skill (Crockett et al., 2006; Magar et al., 2008) although almost all of this has focused on trait characteristics and chronic behavior in wide social contexts. More recently, sport studies have shown interesting, potentially causative links between SR and sporting outcome (Toering and Jordet, 2015) with the impact on practice behaviors as a potentially important mechanism (Toering et al., 2012). Even here, however, the impacts are from trait like behavior to chronic outcomes.

As a parallel development in mainstream psychology, ideas of both MT and SR as potentially transient and variable, state characteristics have emerged. With SR for example, Baumeister and colleagues offer views on the exertion of self-control which “…appears to depend on a limited resource. Just as a muscle gets tired from exertion, acts of self-control cause short-term impairments (ego depletion) in subsequent self-control, even on unrelated tasks” (Baumeister et al., 2006, p. 351). These ideas underpin RDT, which suggests a number of factors such as motivation, personal beliefs and practice as influences against “running out of” SR capacity.

In MT, originally presented solely as a trait, there has been an increasing recognition that it too can vary across situations, once again depending on the presence or absence of certain factors such as personal motivation, belief/expectation and self-efficacy (cf. Gucciardi et al., 2015). There is also a clear need to recognize when to stop trying or turn back. As identified by Crust et al. (2016), accepting one’s own limits and avoiding “costly perseverance” (see also Lucas et al., 2015, p. 606) is an important feature of MT in extreme sports settings. So, for our purposes here, catering for depletion in the short term whilst building resources for the long term emerges as an important psychological focus for P&P coaches and support staff. Furthermore, since depleted self-control effects on skilled task performance have already been shown in laboratory situations (McEwan et al., 2013), this direction of study seemed justified. Before proceeding, however, we should also highlight the extent to which self-control in the present regard is more properly considered as cognitive control (Scherbaum et al., 2018). In simple terms, meeting the action sport challenge is more concerned with the direction of attention against distraction, cognitive control, than resisting temptations – self-control. Reflecting this idea, therefore, we will use SR as pertaining to the optimum direction of cognitive effort.

In addition to these psychological constructs, we should highlight one which has until now been largely used in the physiological sense; the idea of periodization. Originally developed in the former USSR in the 1960s, the idea of designing training programmes to progressively vary load toward a determined peak became a well-established and world wide feature of physical training for athletes (e.g., Bompa, 1984). The approach has undergone a number of reiterations (e.g., Issurin, 2010) but still remains fundamentally unchanged, despite an increasing questioning of the underpinning mechanisms and efficacy of the construct (e.g., Kiely, 2012). Despite these concerns, however, the basic idea of planning the distribution of training inputs to optimize outcome remains both common and indeed, one that is being extended to other elements of training and development such as tactics in team sports (e.g., Tamarit, 2015). In this paper, we suggest another new application of the periodization construct; namely, the systematic variation of emotional load and challenge to optimize the learning and execution of high risk skills.

## Evidence for How These Ideas May Operate – Exemplar Data

If RDT is a genuine factor in P&P skill development, then performers would show development in “bursts” rather than as a steady progression. Notably, however, this pattern would not necessarily be universal, since those “better equipped” on the SR front would cope better and for longer with pressure. Therefore, to really examine for the presence and impact of SR strength, coupled with RDT, an individual focus against tricks of high perceived challenge is necessary. In general, athletes would be expected to show patterns of hard pushing interspersed with slower progress/recovery. Furthermore, from an individual perspective, the push/recover ratio would be expected to vary across athletes depending on their SR skills and mental strength.

Looking at studies of trick progression, for example, this is just what is apparent (cf. Willmott and Collins, 2017). In short, the general push/recover cycle is common across athletes whilst individuals vary in ratio depending on mental skills. Of course, there are undoubtedly a number of other factors which contribute to the “progress in bursts” pattern which is typically apparent. Access to appropriate facilities, including airbags (in some cases) and snow (in most cases!), is just one such pragmatic issue. There are also, however, patterns of development which, we suggest, show a deliberate and carefully planned variation in mental challenge and load reflective of the general and individual factors mentioned above. In this paper we refer to this as “emotional periodization.” For example, athletes getting things set up in phases so that the first attempts of a trick could be timed to meet set dates or attempted in optimum conditions. Often, athletes plan progression into pre-determined time frames in order to achieve sufficient repetition to transition a new trick to specific competitions. Typical catalytic influences being major events such as the X-Games and, more recently, the Olympics. Notably, other periodized plans see trick development focused on optimum conditions, such as the softer and more forgiving snow of a summer training camp.

Coach interviews also show an awareness of the need for periodization (Willmott, 2017, Unpublished). Coaches are very aware of the need to time when to push and when to hold off: making these decisions reliant on a good deal of knowledge about, and carefully developed awareness of, their athletes; an ability to read athletes’ mood early through body language, signs of physical and emotional fatigue, verbal cues, etc. This approach is typically used together with, in many cases, developing the skills to manipulate mood through a variety of subtle and sometimes not so subtle actions, statements and approaches.

Results from athlete surveys (Willmott, 2017, Unpublished) also evidence the emotional periodization approach. Athletes highlight the high emotional effort invested in acquiring new and high-end skills in planning, preparation and execution versus the comparatively straight forward and sometimes *ad hoc* approach to refining existing simpler and/or well rehearsed skills. Of interest, plans are often made for the next day and mental preparation done, with alternatives built in depending on conditions. As one multiple medallist snowboarder stated:

I always go up to the mountain with a plan, right? And I think that’s also key and I’ve seen people get hurt when they are kind of lackadaisical when they go up to the mountain, so I have a plan A and only a plan B maybe a C, right? Because halfpipes are different, weather’s always different.

The point here being that plans were built around the quanta of mental energy needed across the day. This athlete went on to stress the importance of developing then using the mental energy to best effect.

you wanna optimize every single day, …, you wanna make the most of it because you got your coach there you know already going into it mentally you’re like ‘This is a training camp, we gotta get ready, here’s what I wanna do, let’s get after it.

Supporting this view, several athletes talked about the need to build SR strength; to “put money into the bank, then spend it carefully when it will be of most benefit.”

**FIGURE 1:**
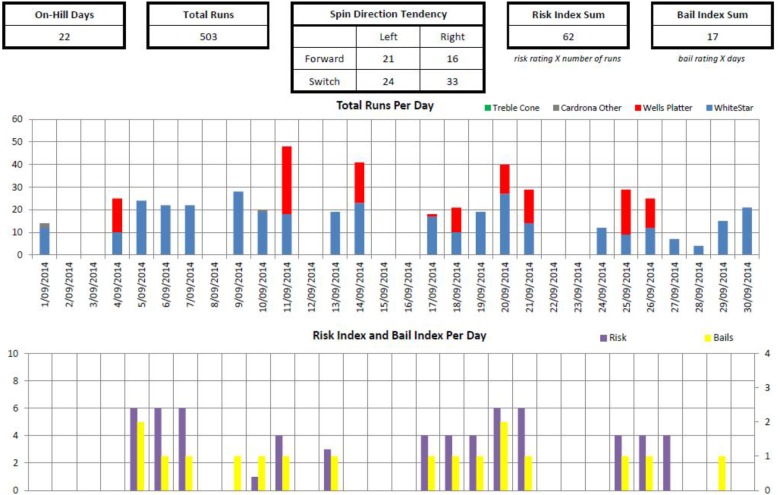
Exemplary data on NZ Team skiers across a training phase.

As a final piece of evidence, we refer to one of the tracking devices developed for use with New Zealand Snowsport athletes. **Figure 1** is an example, covering a 1 month training period.

The pattern of risk shown here makes the point nicely. The athlete “builds up” to a block of high risk/high failure runs (shown in purple and yellow, respectively), takes a break, goes again at a lower level, another break then a peak block of work then a final rest followed by a “consolidation” block to embed the new tricks (cf. Carson and Collins, 2016). As such, we use the physical periodization idea against emotional loading. The figure also shows other ideas relevant for the longer term development of the athlete’s skill base; for example, the need to monitor and work on all spin directions. For the present purpose, however, the periodization of effort is clear, with the athlete building up, working hard at high risk, then taking time to recover in a manner akin to classic concepts of periodization. This pattern is easily apparent when these factors are monitored. As a useful extension to the simple runs per day count provided in **Figure 1**, innovative systems to monitor physical load in P&P are being developed using inertial measuring unit devices to accurately track the number, type, direction and amount of rotations in a training session, along with cumulative landing forces along the lines of previous work in P&P using this type of technology (e.g., Harding et al., 2008; Harding and James, 2010; Scher et al., 2017). Providing useful data on physical indices of loading, other markers impacting emotional loading including “perceived risks taken” and “crashes endured,” complements this data to give a more accurate holistic picture.

## Practical Steps to Counter Negative Influences

Given that emotional periodization is a way in which athletes and coaches can and often do cope with the SR challenges of training and competing in P&P, what methods can be discerned and developed? Given the importance of the coach–athlete relationship (Jowett, 2017), both generally and particularly in such a high-risk domain as P&P, the power dynamic between coach and athlete is a key aspect of SR optimization.

A primary feature of data from both coach and athlete accentuates the coach’s role in empowering athletes via an autonomy-supportive climate (Willmott, 2017, Unpublished; Willmott and Collins, 2017). A key part of the coach role is to help the athlete to accurately assess when to put the hammer down and when to back off. There are several facets to executing this role, including:

- A high level of trust between coach and athlete.
- Guidance from the coach on training load management (both physiological and emotional).
- Coaching awareness of fatigue, fatigue management, and smart decision-making.
- Careful weather forecasting to try to maximize and be ready/recovered for optimal conditions.
- Individual differences: some athletes need to be encouraged and given permission to progress, some athletes need to be given permission to “call it” (finish the session).
- Awareness of the optimal number of repetitions of a risky maneuver to achieve learning growth while avoiding too much fatigue and injury risk.

A blend of classical combined with naturalistic decision-making is apparent in the coaching approach required; an idea developed in action sports by Collins and Collins (2014; 2015; 2016) as Professional Judgment and Decision Making or PJDM (from original work by Martindale and Collins, 2005). All three types of reflection (on-action, on-action-in-context, and in-action) as outlined by Collins and Collins, are cognitive processes in play for the coach.

The above list of elements, either individually or collectively, are reflected by the following selection of quotes from Willmott (2017, Unpublished):

I think it’s important to have that trust with your coach and when I say trust it means they have to be on the same page as you…you have to be vocal with them, let them know how your body’s feeling, um, where your mind’s at.

Male Snowboard Halfpipe Athlete

I didn’t realize how much working on that [new trick] took out of me, then all of a sudden it seemed to hit me, and I was struggling even to do basic stuff. So I think the best thing for me is to take two days off and then get back into it when I’ve recovered and I’m back on my game.

Female Freeski Halfpipe Athlete

It’s a big trick and it’s high risk, it’s day five of the camp and while it’s the last day and we really want to get it done out here, I just think there’s too many red flags. [the athlete] spewed up last night with food poisoning, and he told me he was feeling pretty tired this morning, I think we should work some more into the bag, come away in one piece and come back to taking it to snow another time. What do you think?

Elite Freeski Halfpipe Coach

With more experienced and mature athletes in particular, decision-making can become a joint discussion between athlete and coach, where decisions can be audited and the appropriate outcome agreed:

So I have a confidence I’m like a little scared a little nervous obviously, but when that coach that you have that trust says ‘No dude you’ve got this’ then you’re like ‘OK he’s telling me I got it he can see it from another set of eyes’

Male Snowboard Halfpipe Athlete

In fact, the coach can build emotional periodization into the structure of day-to-day coaching, thus making the need for variation explicit and a normal, accepted part of day-to day work. New Zealand Snowboard coach Sean Thompson has developed a “Push-Drill-Play” structure, which can be used as a daily, weekly or longer element in planning and periodization. For example, each element can be specified in an athlete’s annual plan to describe and differentiate training meso-cycles (4–8 weeks focus), at the micro-cycle level (weeks), or even in terms of a session breakdown. The same approach can be linked to the stages of learning new tricks (cf. Fitts and Posner, 1967). For example, athletes can be asked cto Push at the cognitive stage, to Drill as the skill progresses through the associative stage, then Play as the skill is automated. Further work to embed the skill is completed then returning to Push as the skill is taken to a new level of mastery through further refinement; through combinations into and out of the trick, a grab change, or incorporation in a high level competition run for the first time, for example.

A third factor is the need for athletes to focus on daily recovery mentally as well as physically. Clearly, the impacts involved in P&P can be taxing, whilst activities such as “hiking the pipe” (walking up the side if lift cycles are too long, unavailable or inappropriate) at altitude can make training a physically demanding event. Most of the time though, when generally working with gravity rather than against it, P&P athletes’ energy expenditure and workload is comparatively low (e.g., Zebrowska et al., 2012). As this paper has suggested, however, the emotional challenge can be very high, especially when athletes are taking new tricks to snow for the first time. Accordingly, ensuring sufficient mental recovery is a big feature of life for these athletes. Furthermore, the dynamic nature of the stress–recovery interaction must be considered and catered for (cf. Filho et al., 2015). On a daily basis, for example, coaches and support staff should ensure time away from structured practice and other activities for athletes to decompress. “Vegging out in the hotel room” is an important element of maintaining quality on the hill, not just a mark of idleness! Importance of regular “anchor sleep” is another aspect for attention, whilst the regenerative and learning benefits of sleep are still being realized across sport (cf. Antony et al., 2012).

On a longer-term basis, facilitating engagement in other low-risk but stimulating activities for “re-creation” would be part of the planned process for any training camp. Athletes in most sports get used to living in a close proximity bubble. Getting away from the venue, and indeed each other, is just good sense. Trips for surfing, skating, into different towns or just shopping as “retail therapy” serve to maintain focus on the high-risk days planned. Finally, as a macro concern across the athlete’s career, good practice would encourage life balance and other goals for distraction from the stressors of training and competition; pressures which can be characterized as living life on a knife edge.

Finally, there is a need to address the range of emotional challenges which the athlete encounters, building their skills and confidence to cope proactively (Thatcher et al., 2012) and manipulate emotions for optimum outcomes (cf. Rathschlag and Memmert, 2015). In the present context, arguably the major emotional concern is fear. Of course, fear has a dual role: on one hand it has potential to be the most debilitating emotion to performance, both directly in competition and indirectly by limiting development. On the other hand, it is crucial in terms of informing smart decision-making and keeping an athlete safe. The adrenaline junky idea has been thoroughly discredited – an athlete who feels no fear would not last long in such high-risk environments! Accordingly, one psychological strategy that is more likely to be under the Sport Psychologist’s realm than the coach is the concept of rationalizing fear.

From a psychological perspective, fear has a triple effect. Firstly, it discomforts and changes the focus, making athletes more likely to dwell on and rehearse, either overtly or covertly, making mistakes. This, in turn, increases both the likelihood of occurrence and emotional challenge of attempting the trick (MacPherson et al., 2008). Some (erroneously in our view) see this inhibition as a type of Lost Move Syndrome, or the “Yips” as it is known in Golf (cf. Telegraph, 2014). Thought stopping or relaxation/mindfulness are often the prescription of choice but, since controlling fear whilst staying aware is such a core part of P&P, we would support the development of conscious cognitive control rather than avoidance (cf. Winter et al., 2014), hence our support for Rational Emotive Behavior Therapy as the approach of choice (see below).

Secondly, even if the fear doesn’t actually stop the athlete executing the trick, it can disrupt the timing, placing too much emphasis on one part of the movement. In fact, this can be almost as bad, as the athlete internalizes/embeds a flawed way of doing things which is really hard to clear. Working on this early to build and embed the right rhythm and consequent feel is key here (cf. MacPherson et al., 2009). The use of “video templates,” showing the athlete as self or similar-other model executing at the right pace, is a very useful coaching tool.

As the third challenge, fear exerts a chronic effect, “eating away” at the athlete as s/he struggles to control the intrusive thoughts. Similar to those experienced when returning from injury (e.g., Salim et al., 2016), this pattern can lead to a negative spiral of both acute and chronic disruption. Recognizing that it is the perception of the fear, rather than the arousal itself that is the problem (cf. Raedeke and Stein, 1994), our preferred solution has involved the use of Rational Emotive Behavior Therapy (REBT; Ellis, 1957, 2004; Turner and Barker, 2014). This active approach involves the psychologist exploring, challenging and realigning the emotional reactions of the athlete client. As such, it is perfect for addressing misconceptions or misperceptions which may have occurred but which, if left to persist, may well grow to disproportionate and dysfunctional levels.

## Conclusion

The aim of this paper has been to consider the implications of SR; a major factor for such a mentally demanding high-risk action sport. On the basis of the data cited and discussed, we would suggest that P&P athletes could usefully be surveyed and compared to the extreme mountaineers examined by Crust et al. (2016), not least for the similarity that too much MT in action sports (especially without enough experience) can result in injury or even death through impaired decision making. Data are clearly supportive of a short term, transient and context-specific type of MT, through which athletes make informed decisions about the acceptability of risk. This awaits further work and is highlighted as an area for further investigation.

From an applied perspective, we have listed several steps and procedures through which emotional pressures can be monitored, controlled for and addressed. The use of mental skills training as an adjunct to these ideas is another important feature of the modern P&P experience (cf. Willmott and Collins, 2015). As such, work here is paralleling but also extending in depth and range, work on psychological skills training in other challenging domains (e.g., High Intensity Sports; Birrer and Morgan, 2010). Investment in skills development is often seen as a longer term, even career long factor. In our experience, however, much can be achieved through short, intensive and challenge-specific interventions. Certainly positive changes can be affected in relevant skills with short term intense interventions (e.g., 5 days of meditation; Tang et al., 2007). The optimum use of support specialists is another topic for further investigation. For the present, however, the importance of optimizing SR and MT in P&P athletes is an important applied issue and also one with a sound theoretical grounding.

## Ethics Statement

This study was carried out in accordance with the recommendations of University of Central Lancashire. The protocol was approved by the BAHSS ethics committee. All subjects gave written informed consent in accordance with the Declaration of Helsinki.

## Author Contributions

DC contributed to the conception of the study initial writing and review of the manuscript and analysis. TW contributed in data collection and analysis, plus conception of study, and writing of the manuscript. LC contributed to analysis, review, and writing of the manuscript.

## Conflict of Interest Statement

The authors declare that the research was conducted in the absence of any commercial or financial relationships that could be construed as a potential conflict of interest.
